# Aclacinomycin A Sensitizes K562 Chronic Myeloid Leukemia Cells to Imatinib through p38MAPK-Mediated Erythroid Differentiation

**DOI:** 10.1371/journal.pone.0061939

**Published:** 2013-04-17

**Authors:** Yueh-Lun Lee, Chih-Wei Chen, Fu-Hwa Liu, Yu-Wen Huang, Huei-Mei Huang

**Affiliations:** 1 Department of Microbiology and Immunology, College of Medicine, Taipei Medical University, Taipei, Taiwan; 2 Neurosurgery Department, Chi Mei Medical Center, Tainan, Taiwan; 3 Institute of Molecular Biology, Academia Sinica, Taipei, Taiwan; 4 Graduate Institute of Medical Sciences, College of Medicine, Taipei Medical University, Taipei, Taiwan; Baylor College of Medicine, United States of America

## Abstract

Expression of oncogenic Bcr-Abl inhibits cell differentiation of hematopoietic stem/progenitor cells in chronic myeloid leukemia (CML). Differentiation therapy is considered to be a new strategy for treating this type of leukemia. Aclacinomycin A (ACM) is an antitumor antibiotic. Previous studies have shown that ACM induced erythroid differentiation of CML cells. In this study, we investigate the effect of ACM on the sensitivity of human CML cell line K562 to Bcr-Abl specific inhibitor imatinib (STI571, Gleevec). We first determined the optimal concentration of ACM for erythroid differentiation but not growth inhibition and apoptosis in K562 cells. Then, pretreatment with this optimal concentration of ACM followed by a minimally toxic concentration of imatinib strongly induced growth inhibition and apoptosis compared to that with simultaneous co-treatment, indicating that ACM-induced erythroid differentiation sensitizes K562 cells to imatinib. Sequential treatment with ACM and imatinib induced Bcr-Abl down-regulation, cytochrome c release into the cytosol, and caspase-3 activation, as well as decreased Mcl-1 and Bcl-xL expressions, but did not affect Fas ligand/Fas death receptor and procaspase-8 expressions. ACM/imatinib sequential treatment-induced apoptosis was suppressed by a caspase-9 inhibitor and a caspase-3 inhibitor, indicating that the caspase cascade is involved in this apoptosis. Furthermore, we demonstrated that ACM induced erythroid differentiation through the p38 mitogen-activated protein kinase (MAPK) pathway. The inhibition of erythroid differentiation by p38MAPK inhibitor SB202190, p38MAPK dominant negative mutant or p38MAPK shRNA knockdown, reduced the ACM/imatinib sequential treatment-mediated growth inhibition and apoptosis. These results suggest that differentiated K562 cells induced by ACM-mediated p38MAPK pathway become more sensitive to imatinib and result in down-regulations of Bcr-Abl and anti-apoptotic proteins, growth inhibition and apoptosis. These results provided a potential management by which ACM might have a crucial impact on increasing sensitivity of CML cells to imatinib in the differentiation therapeutic approaches.

## Introduction

Chronic myeloid leukemia (CML) is a malignant hematological disease of hematopoietic stem/progenitor cells characterized by the presence of Bcr-Abl oncoprotein, a constitutively active tyrosine kinase produced as a reciprocal translocation between chromosome 9 and 22 [1]. Bcr-Abl blocks cell differentiation and protects cells from apoptosis to allow the proliferation of undifferentiated stem cells in the absence of growth factors [2]. CML progenitor cells undergo excess proliferation during chronic phase, these cells still maintain the capacity to differentiate and function normally. Ultimately, CML progresses from the chronic phase to the blast crisis phase, in which differentiation becomes arrested. This results in the accumulation of undifferentiated CML progenitors in bone marrow and peripheral blood [3], [4].

A specific inhibitor of Bcr-Abl tyrosine kinase, imatinib (STI571 or Gleevec), is highly effective in treating CML patients, and is used as first-line treatment for CML [3], [4]. However, chronic phase CML develop imatinib resistance after prolong treatment, and patients with accelerated phase or blast crisis phase CML become resistant to imatinib treatment [5], [6], [7]. Therefore, the development of other strategies is needed to treat this disease. In recent years, special attention has focused on a small population of CML stem cells which may contribute to the pathogenesis of relapse and therapeutic resistance [8], [9]. Differentiation therapy is now regarded as a promising therapeutic approach.

Anthracyclines such as doxorubicin and aclacinomycin A (ACM), also known as aclarubicin, are effective antitumor antibiotics. ACM is a class of microbial secondary metabolites produced by Streptomyces galilaeus. ACM has been widely used in the clinic to treat various cancers [10], [11], [12]. ACM cytotoxic effect is due to its DNA intercalating activity [13]. In addition, ACM interacts with topoisomerase I and II, and acts as an inhibitor of these enzymes [14], [15]. In addition to its cytotoxic effect, a low concentration of ACM has shown to induce erythroid specific gene expressions and erythroid differentiation of human CML cell line K562 [16], [17]. We hypothesize that ACM induction of cell differentiation could sensitize K562 CML cells to imatinib.

K562 is a hematopoietic progenitor cell line established from a human CML patient in blast crisis [18], [19]. K562 cells possess the capability of unlimited proliferation, but are unable to proceed with differentiation. Previous studies indicated that p38 mitogen-activated protein kinase (MAPK) played an important role in inducing erythroid differentiation of hematopoietic progenitor cells and CML cells [20], [21], [22]. However, the role of p38MAPK in ACM-mediated erythroid differentiation has not been explored. Thus, in the current study, we evaluated whether ACM can sensitize K562 cells to imatinib. We also explored whether p38MAPK is involved in ACM-induced erythroid differentiation, and whether the blockage of p38MAPK activity leads to the decreased ACM induction of erythroid differentiation and ACM-induced sensitization of K562 cells to imatinib.

## Materials and Methods

### Materials

3-(4,5-Dimethylthiazol-2-yl)-2,5-diphenyltetrazolium bromide (MTT), benzidine, SB202190, anti-Flag antibody, arachidonic acid (AA) and U0126 were purchased from Sigma (St. Louis, MO). Aclacinomycin A (ACM) was purchased from Enzo Life Sciences (San Diego, CA). Imatinib was kindly provided by Novartis Pharma AG (Basel, Switzerland). Antibodies for Western blotting, including voltage dependent anion channel (VDAC), procaspase-9, procaspase-8, procaspase-3, cleaved caspase-3, PARP, Bcl-xL, phospho-p38MAPK, p38MAPK, and phospho-ATF2 were obtained from Cell Signaling Technology (Danvers, MA). Antibodies specific for c-Abl, cytochrome c, Mcl-1, Fas, Fas ligand, and β-actin were purchased from Santa Cruz Biotechnology (Santa Cruz, CA). The caspase-3 inhibitor (z-DEVD-fmk) and caspase-9 inhibitor (z-LEHD-fmk) were obtained from BD Biosciences.

### Cell culture

The human CML cell line K562 was purchased from Bioresource Collection and Research Center (BCRC) of Taiwan (BCRC 60007) and cultured in RPMI-1640 medium supplemented with 10% fetal bovine serum (FBS), 4 mM L-glutamine, 100 U/ml penicillin, and 100 µg/ml streptomycin in a 5% CO_2_ incubator at 37°C. K562/p38α(AF)1 cells stably expressing a dominant-negative form (AF) of p38α [p38α(AF)] in K562 cells were established previously [23]. K562/p38α(AF)1 cells were maintained in the same medium as parental K562 cells, except for the inclusion of 200 µg/ml of G418 in the medium.

### MTT assay

K562 cells were seeded into a 96-well plate at a density of 1.25×10^4^ cells/ml per well in 200 µL of complete medium and treated under specified scheme. After 6 days of incubation, MTT (10 µl, 5 mg/ml in PBS) was added to each well and incubated for 3–4 hours. The MTT solution was removed from the wells by aspiration and the formazan crystals were dissolved in DMSO (100 µl). Absorbance was measured at 570 nm using a model 450 microplate reader (BIO-RAD Laboratories).

### Benzidine staining assay

Erythroid differentiation was determined by hemoglobin synthesis in K562 cells using a benzidine staining assay as previously described [24]. Briefly, cells were cultured in the indicated medium at a density of 1×10^5^ cells/ml for 3 days. Cells were suspended in a staining solution of 49∶1 ratio of benzidine solution (0.2% benzidine in 0.5% acetic acid) to 30% H_2_O_2_, and then subjected to cytospin centrifugation after 10 minutes of incubation at room temperature. The black benzidine-stained hemoglobin-positive cells were determined microscopically. At least 300 cells were counted in triplicate for each condition.

### Annexin V/propidium iodide (PI) staining and flow cytometry

The level of cell apoptosis was measured by annexin V-FITC and PI staining. Cells were cultured in the indicated scheme for the specified time, collected by centrifugation, and washed with PBS. Cells were stained with annexin V-FITC and PI (Invitrogen) and incubated for 15 min at room temperature in the dark. Samples of 10^4^ cells for each scan were acquired on a FACScan flow cytometer (Becton Dickinson, Mountain View, CA), and analyzed with Cellquest software (Becton Dickinson). Results are shown as the percentage of early (annexin V^+^ PI^−^) and late apoptotic cells (annexin V^+^ PI^+^) [24].

### Preparation of cytosolic fractions and assessment of cytochrome c release

After washing with PBS, cell pellets were resuspended in 200 µl of buffer [20 mM HEPES-KOH (pH 7.5), 10 mM MgCl_2_, 1 mM EDTA, 1 mM EGTA, 1 mM DTT, 250 mM sucrose and proteinase inhibitors]. After homogenization, unbroken cells, large plasma membranes, and nuclei were removed by centrifugation at 1000 *g* for 1 min at 4°C. The supernatant was centrifuged at 10,000 *g* for 20 min at 4°C. The supernatant was additionally centrifuged at 50,000 *g* for 2 h to obtain the cytosolic fraction. The cytosolic protein samples were quantified and subjected to Western blot analysis.

### Western blot analysis

After washing with PBS, cells were lysed at 4°C in lysis buffer [1% Triton X-100, 20 mM Tris (pH 7.5), 150 mM NaCl, 1 mM EGTA, 1 mM EDTA, 2.5 mM sodium pyrophosphate, 1 mM β-glycerolphosphate, 1 mM PMSF, 1 µg/ml leupeptin, and 1 mM Na_3_VO_4_]. The non-soluble lysates were removed by centrifugation. Protein lysates (30 µg) were resolved using sodium dodecylsulfate polyacrylamide gel electrophoresis (SDS-PAGE), the protein bands transferred to polyvinylidene difluoride (PVDF) membranes (Millipore, Bedford, MA), then the membrane probed with primary antibodies. After binding with horseradish peroxidase-conjugated secondary antibodies, the blots were visualized with an enhanced chemiluminescence (ECL) detection system (PerkinElmer Life and Analytical Sciences, Waltham, MA). Quantification of protein level was performed using a computing densitometer with scientific imaging systems (Kodak, Rochester, NY, U.S.A.).

### In vitro kinase assay

The p38MAPK in vitro kinase assay was performed using the p38MAPK kinase assay kit (Cell Signaling Technology). Briefly, total cell extracts were prepared as described [23], and p38kinases were immunoprecipitated with the immobilized phospho-p38MAPK monoclonal antibody. After washing twice with lysis buffer and twice with kinase buffer (25 mM Tris-HCl, pH7.5, 5 mM β-glycerolphosphate, 2 mM DTT, 0.1 mM Na_3_VO_4_, 10 mM MgCl_2_), the immunoprecipitates were assayed for p38MAPK kinase activity in kinase buffer with 200 mM ATP and 2 mg ATF2 fusion protein per reaction. The reaction was stopped with SDS sample buffer and analyzed by Western blotting with specific anti-phospho-ATF2 antibody.

### Knockdown of p38MAPK by short hairpin RNAs (shRNAs)

K562 cells (1×10^6^) were mixed with 100 µl of Nucleofector solution V (Amaxa Biosystems) and 4 µg of SureSilencing short hairpin RNA (shRNA) plasmids. Transfection was carried out using the Nucleofector apparatus (Amaxa Biosystems). The program T-16 was used for transfection. SureSilencing shRNA plasmids for human p38α (5′-CAAGGTCTCTGGAGGAATTCA-3′) and a negative control shRNA plasmid (no. KH01361) were purchased from SABioscience Corporation (Frederick, MD).

### Statistics

Quantitative data are presented as the mean and standard error of the mean (SEM). Statistically significant differences between groups were analyzed with Student’s *t*-test. A *p* value of<0.05 was considered significant.

## Results

### Optimal concentration of ACM induced cell differentiation but did not affect cell growth and apoptosis in K562 cells

**Figure 1:**
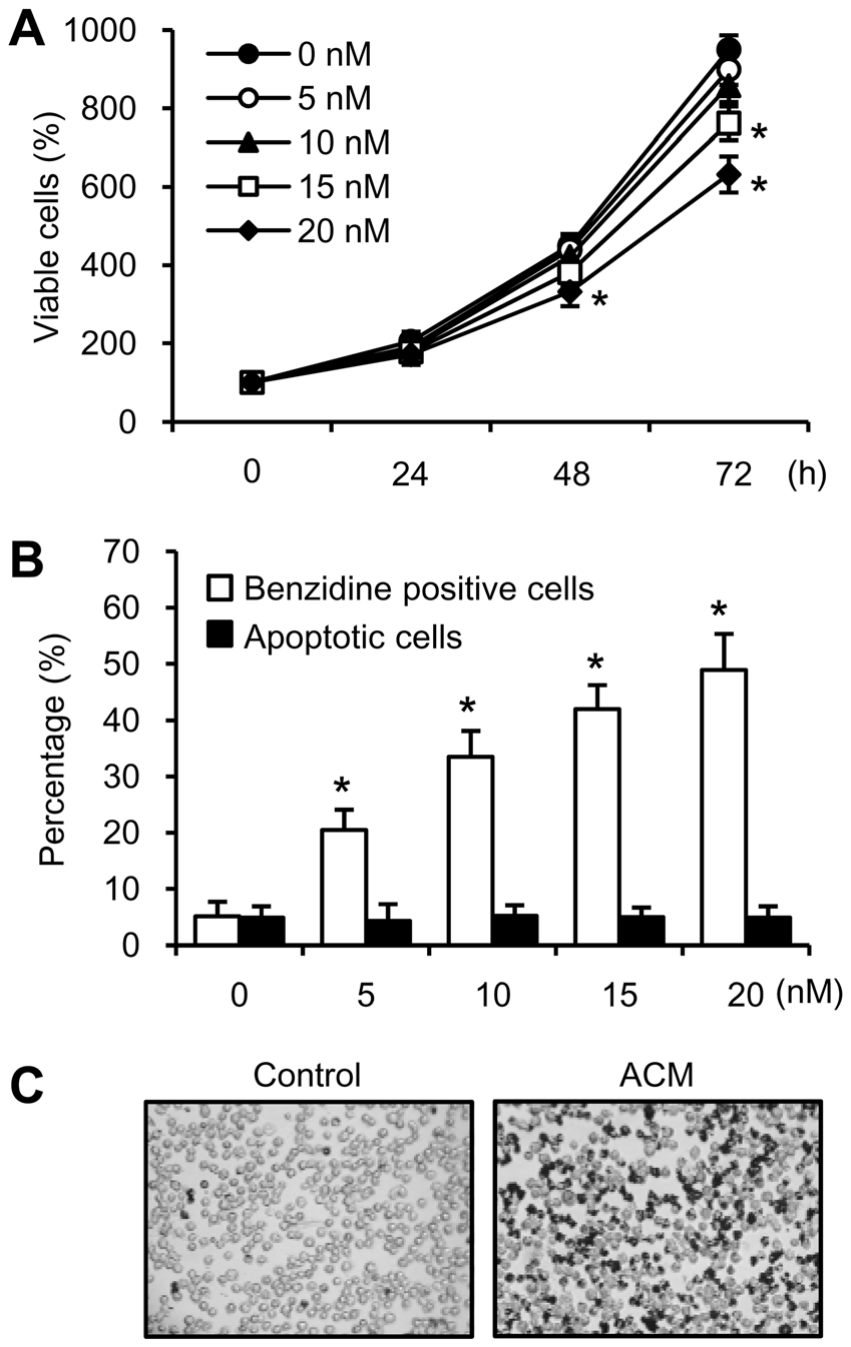
Determination of the optimal concentration of ACM for erythroid differentiation but not growth inhibition and apoptosis. (A) Cells were treated with or without ACM (5, 10, 15 or 20 nM) for 24 to 72 hours. Cell viability was detected by MTT assay. (B) Intracellular hemoglobin was detected by benzidine staining assay. Apoptotic cells were stained with annexin V-FITC and PI and analyzed by flow cytometry. Values are mean ± SEM from three experiments. ^*^, *p*<0.05, *versus* untreated control (A and B). (C) K562 cells were treated without (control) or with 10 nM ACM for 3 days and then analyzed with benzidine staining assay.

K562 cells can be induced to differentiate towards erythroid lineage after exposure to ACM [16], [17]. In order to investigate whether ACM-induced differentiation can increase sensitivity of K562 cells to imatinib, we first determined the concentration of ACM required for erythroid differentiation but not growth inhibition and apoptosis. Cells were exposed to ACM and the cell viability was evaluated by MTT assay. The treatment of K562 cells for 24 to 72 h with 5, 10, 15 and 20 nM of ACM resulted in cell growth inhibition in a dose- and time-dependent manner. ACM at 15 nM significantly decreased cell viability in K562 cells after 72 h treatment. ACM at 20 nM also significantly decreased cell viability in K562 cells after 48 and 72 h treatments (Figure 1A). The differentiating cells produce hemoglobin, an erythroid marker, which can be stained positively with benzidine. K562 cells were exposed to increasing concentrations of ACM (5–20 nM) for 72 hours. Figure 1B shows that treatment with ACM increased the percentage of benzidine positive cells in a dose-dependent manner. ACM (5–20 nM) did not induce apoptosis in K562 cells (Fig. 1B). Figure 1C shows the number of hemoglobinized cells was increased by 10 nM ACM compared to the untreated control, and analyzed by benzidine staining assay. In order to induce cell differentiation but not growth inhibition and apoptosis, the 10 nM of ACM was then used to investigate the relationship between cell differentiation and imatinib sensitivity in the following experiments.

### ACM induction of erythroid differentiation sensitized K562 cells to a minimally toxic concentration of imatinib

**Figure 2:**
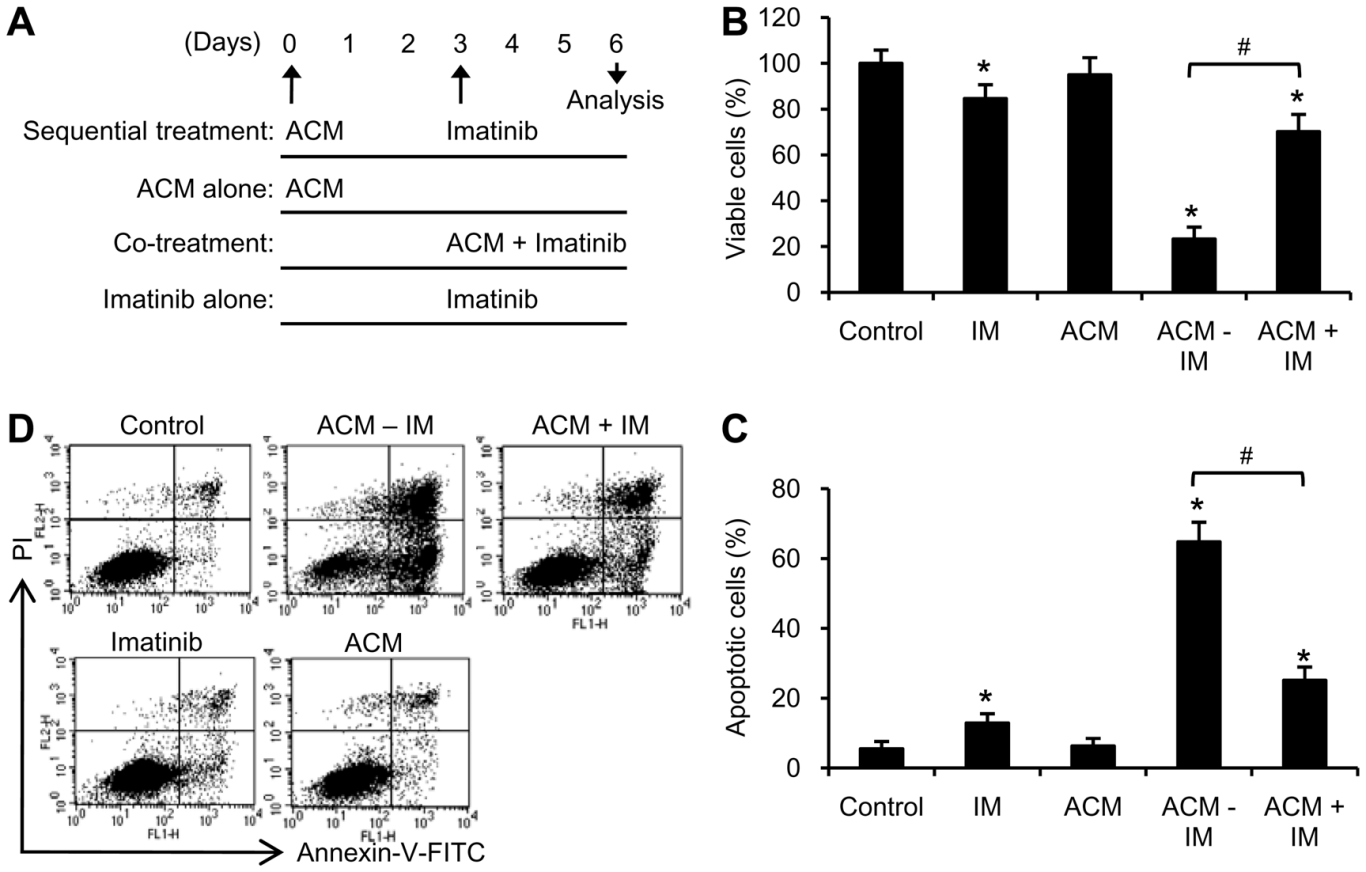
Sequential treatment with ACM and imatinib strongly induced growth inhibition and apoptosis in K562 cells. (A) Treatment scheme for ACM and imatinib in K562 cells. Sequential treatment (SE): cells were treated with 10 nM ACM for 3 days and then with 200 nM imatinib for additional 3 days (ACM-IM). ACM alone treatment: cells were treated with 10 nM ACM for 6 days. Co-treatment (CO): cells were simultaneously treated with 10 nM ACM and 200 nM imatinib for 3 days (ACM+IM). Imatinib alone treatment (IM): cells were treated with 200 nM imatinib for 3 days. (B) K562 cells (1.25×10^4^ cells/ml) were seeded in 96 well plate (200 µl per well) and treated as described above. Cell viability was analyzed by MTT assay. (C) Cells (1.25×10^4^ cells/ml) were seeded in 6 well plate (3 ml per well) and treated as described in Figure 2A. Apoptotic ells were stained with annexin V-FITC and PI and analyzed by flow cytometry. Values are mean ± SEM from four experiments. ^*^, *p*<0.05 *versus* untreated control. ^#^, *p*<0.05 (B and C). (D) Flow cytometry data show representative results from one of four independent experiments.

In order to investigate whether ACM-induced differentiation can increase sensitivity of K562 cells to imatinib, cells were treated under the following conditions: (i) sequential treatment with ACM and imatinib; (ii) co-treatment with ACM and imatinib; (iii) treatment with ACM alone; (iv) treatment with imatinib alone (Fig. 2A). Treatment with 200 nM imatinib slightly inhibited cell viability (Fig. 2B), and slightly induced apoptosis (Fig. 2C, D). Simultaneous co-treatment with 10 nM ACM and 200 nM imatinib reduced cell viability (70.2%±7.5% viability, Fig. 2B) and increased cell apoptosis (25.2%±3.8%, Fig. 2C, D) compared with the untreated control in K562 cells. However, sequential treatment with ACM followed by imatinib strongly decreased cell viability (23.3%±5.2% viability, Fig. 2B) and strongly increased apoptosis (65%±5.6% apoptosis, Fig. 2C, D). These results suggest that differentiated K562 cells are sensitive to a minimally toxic concentration of imatinib.

### Sequential treatment with ACM and imatinib down-regulated Bcr-Abl, Mcl-1 and Bcl-xL, as well as activated caspase-3

**Figure 3:**
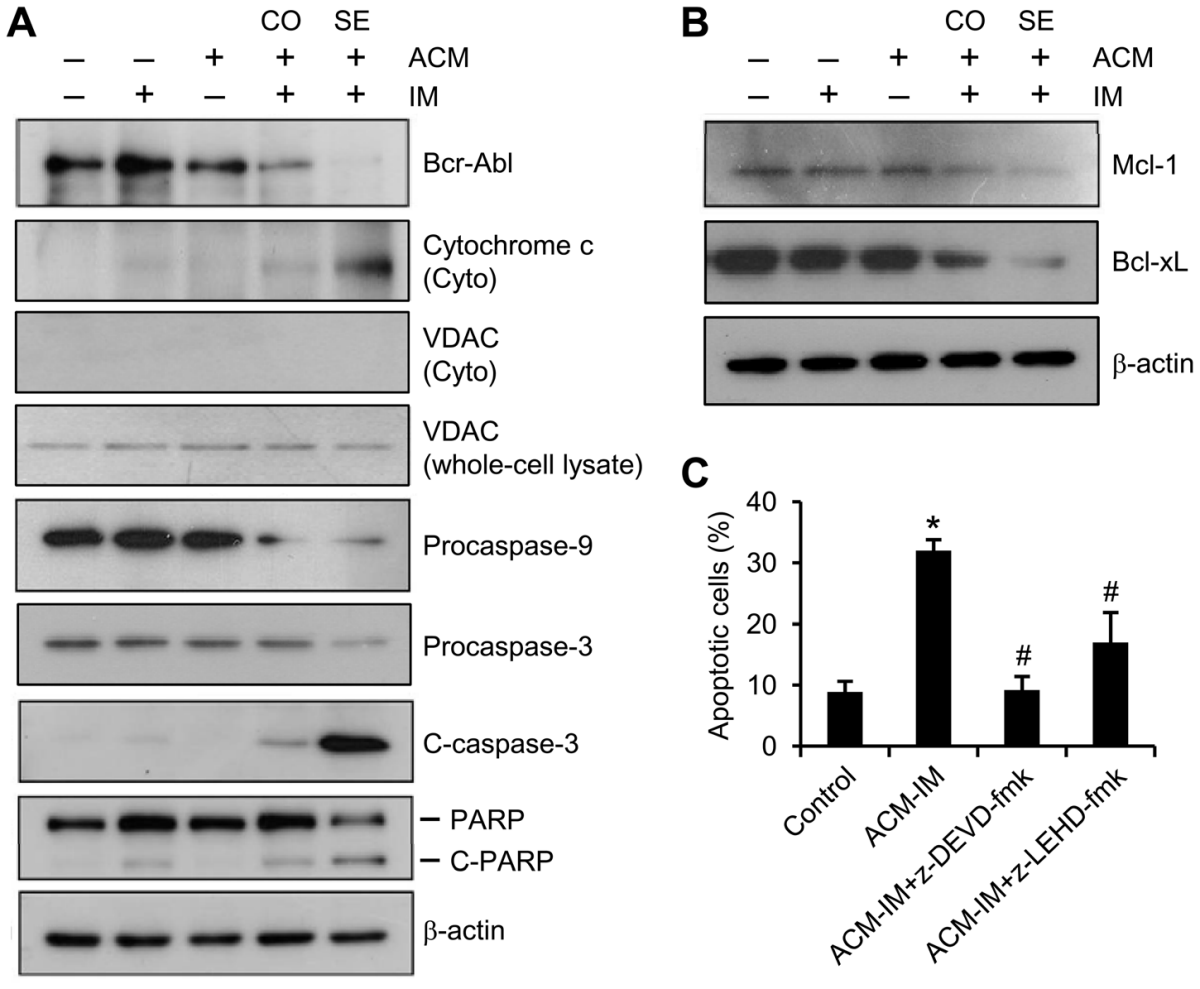
Sequential treatment with ACM and imatinib down-regulated Bcr-Abl, Mcl-1 and Bcl-xL, and activated caspase cascade. (A and B) Cells were treated as described in Figure 2A. Cells were harvested and lysed, and the proteins were subjected to Western blot analysis using specific antibodies against Bcr-Abl, cytochrome c, VDAC, procaspase-9, procaspase-3, cleaved caspase-3 (C-caspase-3), PARP, Mcl-1 and Bcl-xL. β-actin was used as loading control. (C) K562 cells we treated with 5 nM ACM for three days. Subsequently they were treated for an additional 3 days with either: a. 200 nM imatinib (ACM-IM), b. 200 nM imatinib and 50 µM caspase-3 inhibitor (ACM-IM+z-DEVD-fmk), or c. 200 nM imatinib and 100 µM caspase-9 inhibitor (ACM-IM+z-LEHD-fmk). Apoptotic ells were stained with annexin V-FITC and PI and analyzed by flow cytometry. Values are mean ± SEM from three experiments. ^*^, *p*<0.05 *versus* untreated control. ^#^, *p*<0.05 *versus* ACM-IM group.

To reveal the mechanism of the sequential treatment of ACM and imatinib-induced apoptosis in the K562 CML cell line, we analyzed expression of the Bcr-Abl protein by Western blotting. No significant reduction in the expression level of Bcr-Abl was observed following treatment with either agent alone compared to the untreated control (Fig. 3A). Whereas the combination treatment of 10 nM ACM with 200 nM imatinib yielded mild responses, the sequential treatment scheme resulted in striking decrease in the expression levels of Bcr-Abl and increase in release of cytochrome c into the cytosol. The cytosolic fraction was checked for purity by Western blotting using VDAC as a mitochondrial marker. No contamination of the VDAC was observed in the cytosolic fraction in K562 cells (Fig. 3A). These sequential treatment effects were accompanied by marked decrease in procaspase-9 and procaspase-3, and increase in caspase-3 cleavage product and degradation of PARP (Fig. 3A). Although imatinib alone treatment had slight effects on caspase-3 cleavage and PARP degradation, combined treatment with ACM and imatinib increased the effects mildly. Since ACM/imatinib sequential treatment significantly increased apoptosis and activated caspase-3, we analyzed the levels of anti-apoptotic proteins, Mcl-1 and Bcl-xL, in K562 cells (Fig. 3B). Combined treatment with ACM and imatinib induced a decrease in expression of Mcl-1 and Bcl-xL, whereas sequential treatment resulted in a further decrease in Mcl-1 and Bcl-xL expressions (Fig. 3B). In addition, it is important to know whether apoptosis induced by ACM/imatinib sequential treatment was affected by the caspase-9 inhibitor (z-LEHD-fmk) and caspase-3 inhibitor (z-DEVD-fmk). We found that z-LEHD-fmk and z-DEVD-fmk both suppressed ACM/imatinib sequential treatment-induced apoptosis in K562 cells (Fig. 3C), thus confirming the participation of caspase-9 and caspase-3.These results indicate that pretreatment of K562 cells with ACM followed by a subtoxic concentration of imatinib resulted in a marked decrease in the expressions of Bcr-Abl and anti-apoptotic proteins and increase in the activation of caspase cascade.

### Sequential treatment with ACM and imatinib induced apoptosis independent of Fas receptor system

**Figure 4:**
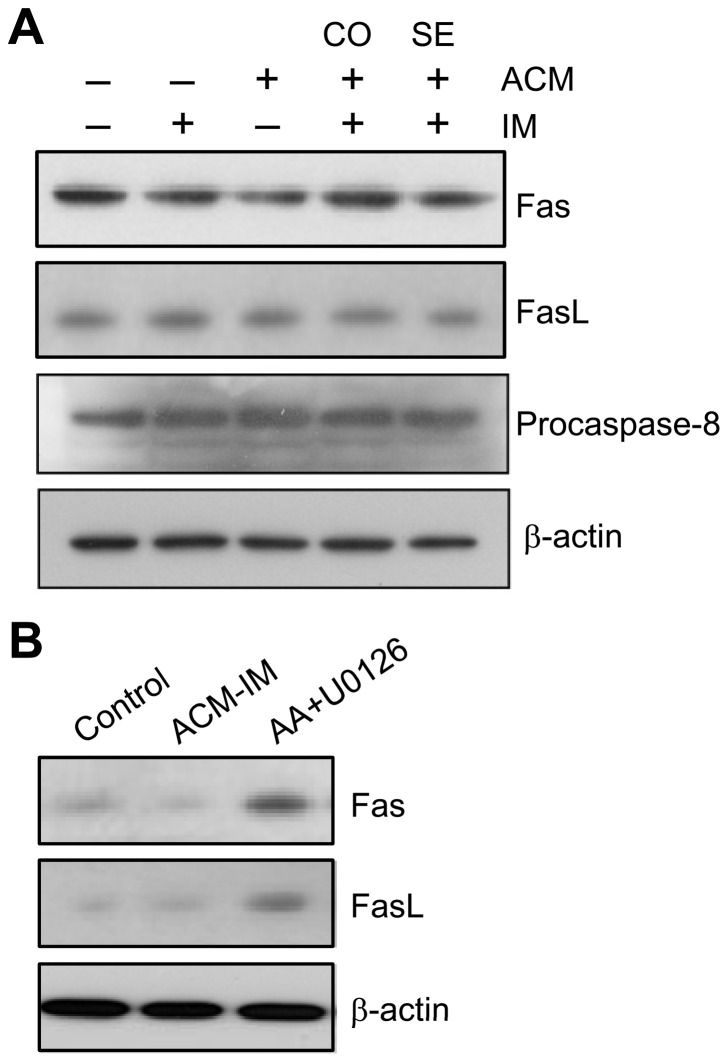
Sequential treatment with ACM and imatinib did not affect the Fas receptor system. (A) Cells were treated as described in Figure 2A. Cells were harvested and lysed, and the proteins were subjected to Western blot analysis using specific antibodies against Fas, Fas ligand (FasL) and procaspase-8. β-actin was used as loading control. (B) Cells were co-treated with AA and U0126 for 24 h. Western blot analyses of Fas and Fas ligand in ACM-IM-treated cells and AA+U0126-treated cells.

Apoptosis may also be induced by the Fas receptor system [25]. As shown in Figure 4A, the Fas receptor expression level was unchanged in the K562 cells after ACM/imatinib sequential treatment compared with untreated control. The Fas ligand expression level was not increased with sequential treatment scheme. Fas ligand binds to Fas receptor that leads to caspase-8 cleavage and activation [25]. The expression level of procaspase-8 was also unchanged in the K562 cells after ACM/imatinib sequential treatment (Fig. 4A). Previous studies showed that a combination of arachidonic acid (AA) and U0126 can increase the protein level of Fas/Fas ligand in K562 cells [26]. The addition of AA and U0126-increased Fas/Fas ligand was used to provide for a positive control to make sure the level of Fas and Fas ligand indeed can be altered in K562 cells (Fig. 4B).

### p38MAPK activation was involved in ACM-induced erythroid differentiation of K562 cells

**Figure 5:**
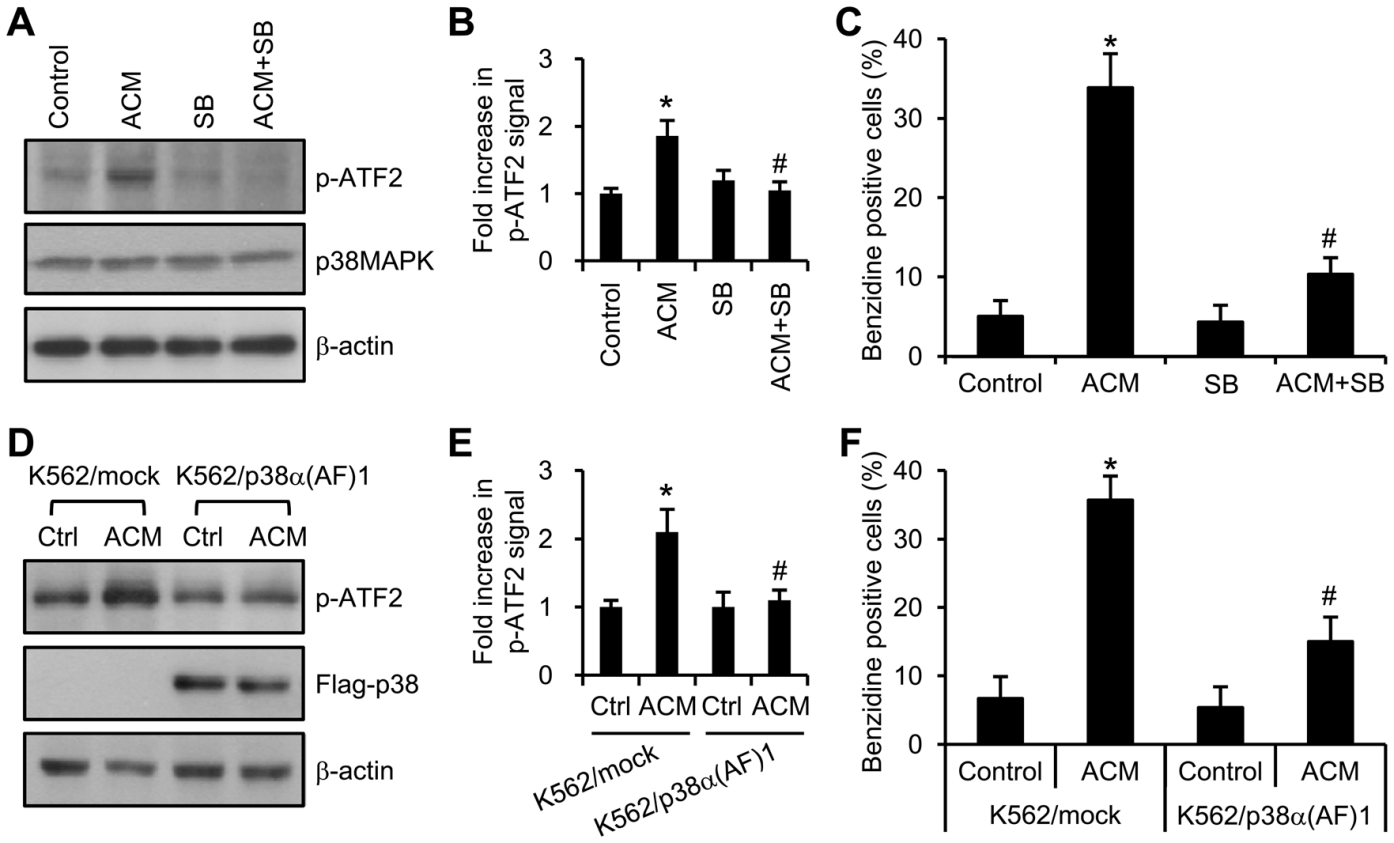
ACM induced erythroid differentiation of K562 cells through p38MAPK pathway. (A) K562 cells were treated with or without (control) 10 nM ACM, 2 µM SB202190 (SB), or ACM plus SB202190 for 3 days. Cell lysates were immunoprecipitated with anti-phospho-p38MAPK antibody. The immunoprecipitates were then subjected to in vitro kinase assay for p38MAPK described in ‘‘Materials and Methods’’. Phospho-ATF2 (p-ATF2) is the product of the kinase reaction determined by Western blotting using anti-p-ATF2 antibody. P38MAPK and β-actin were used as loading controls. Experiments were repeated three times independently. (B) Values of fold increase in p-ATF2 signal are means ± SEM. ^*^, *p*<0.05 *versus* untreated control. ^#^, *p*<0.05 *versus* ACM treatment. (C) K562 cells were treated as described in panel A. Hemoglobin production was detected by benzidine staining assay. (D) K562/mock and K562/p38α(AF)1 cells were treated with or without (control) 10 nM ACM for 3 days. Activation of p38MAPK was measured by in vitro kinase assay. The expression of p38α(AF) was confirmed by Western blotting using anti-Flag antibody. β-actin was used as loading control. Experiments were repeated three times independently. (E) Values are means ± SEM. ^*^, *p*<0.05 *versus* K562/mock-control. ^#^, *p*<0.05 *versus* K562/mock + ACM. (F) K562 cells were treated as described in panel D. Intracellular hemoglobin was detected by benzidine staining assay.

ACM induces erythroid differentiation of K562 cells. However, the mechanism remains unknown. Previous studies showed that p38MAPK regulates the erythroid differentiation of hematopoietic progenitor cells and CML cells [20], [21], [22]. We next to study whether ACM induces erythroid differentiation through p38MAPK pathway; and the inactivation of p38MAPK could inhibit ACM/imatinib sequential treatment-mediated growth inhibition and apoptosis. The contribution of the p38MAPK in ACM-induced erythroid differentiation was determined by the results obtained with a specific inhibitor of p38MAPK, SB202190, and with a p38MAPK dominant negative mutant. The kinase activity for p38MAPK was measured by in vitro kinase assay. A known p38MAPK substrate, ATF-2, was included in the p38MAPK kinase reaction and its phosphorylation was detected with phospho-ATF-2 specific antibody. Treatment of K562 cells with SB202190 inhibited ACM-stimulated p38MAPK kinase activity (Fig. 5A,B) and hemoglobin synthesis (Fig. 5C). Our previous studies have established K562/p38α(AF)1 cells stably expressing dominant negative mutant of p38MAPK, with no p38MAPK kinase activity in K562 cells [23]. Figure 5D and E show that dominant negative mutant of p38MAPK, p38α(AF), was able to significantly block ACM-activated p38MAPK kinase activity, and reduced the ACM induction of hemoglobin synthesis in K562/p38α(AF)1 cells (Fig. 5F). The results described above suggest that p38MAPK has an important role in ACM-induced erythroid differentiation.

### Sequential treatment with ACM and imatinib-induced growth inhibition and apoptosis which were reduced by inhibition of the p38MAPK pathway

**Figure 6:**
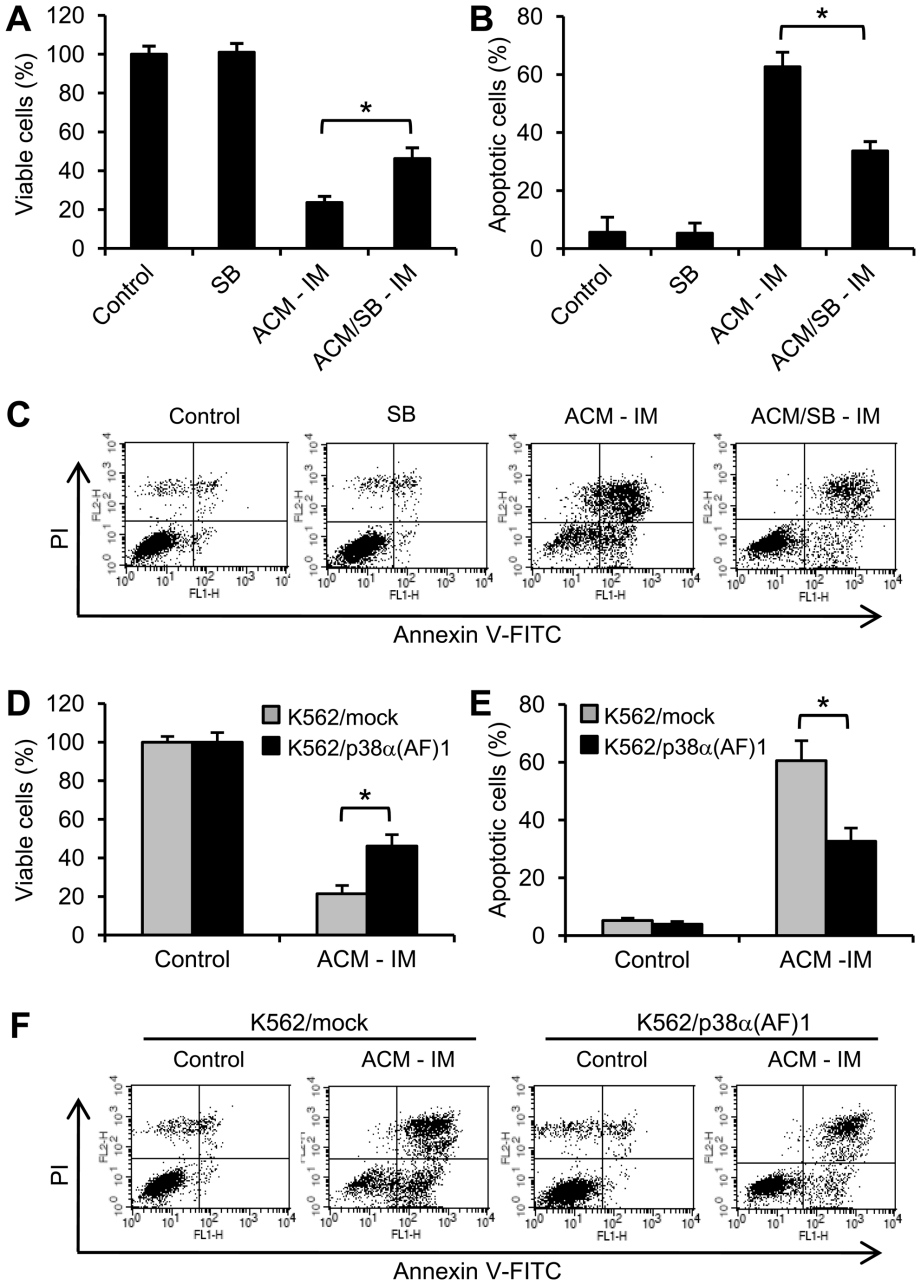
Sequential treatment with ACM and imatinib-induced growth inhibition and apoptosis were regulated by p38MAPK pathway. (A) K562 cells were pretreated with 10 nM ACM in combination without or with 2 µM SB202190 (SB) for 3 days and then with 200 nM imatinib for additional 3 days (ACM-IM or ACM/SB-IM). (B) Cells were treated as described in Figure 6A. (C) Flow cytometry data (Fig. 6B) show representative results from one of four independent experiments. (D) K562/mock and K562/p38α(AF)1 cells were treated per sequential treatment scheme as described in Figure 2A. (E) Cells were treated as described in Figure 6D. (F) Flow cytometry data (Fig. 6E) show representative results from one of four independent experiments. (A and D) Cell viability was analyzed by MTT assay. (B and E) Apoptotic ells were stained with annexin V-FITC and PI and analyzed by flow cytometry. Values are mean ± SEM from four experiments. ^*^, *p*<0.05 (A, B, D and E).

**Figure 7:**
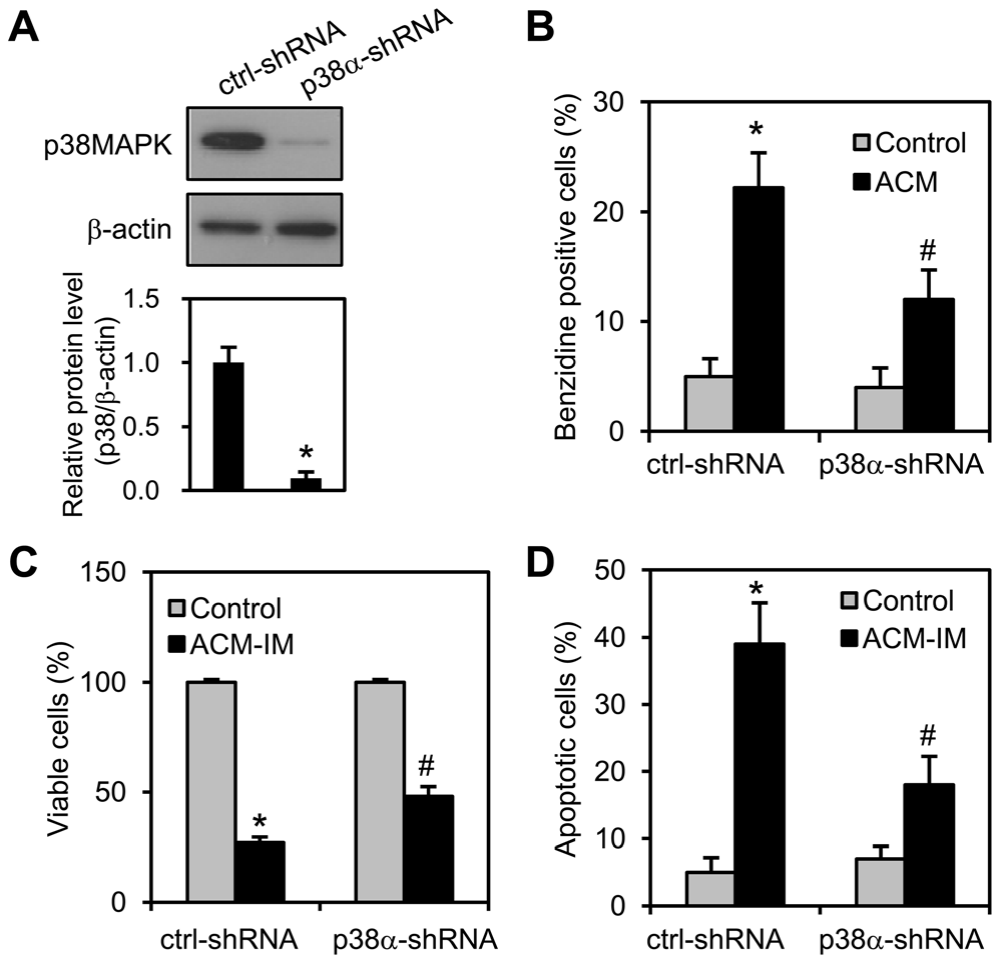
The knockdown of p38MAPK and reduction of erythroid differentiation decreased K562 cell sensitivity to imatinib. The cells were transfected with a negative control shRNA (ctrl-shRNA) or a shRNA targeting p38α (p38α-shRNA) for 3 days. (A) The protein level of p38MAPK was analyzed by Western blotting (upper panel). Immunoblots are representative of three experiments, which are presented as the mean ± SEM (lower panel). ^*^, *p*<0.05 *versus* control-shRNA. (B) K562 cells were transfected with shRNA plasmids and treated without (control) or with ACM (5 nM). Cells were stained with benzidine to determine hemoglobin synthesis at 72 h. (C) The shRNA-transfected cells were treated with ACM (5 nM) for 3 days and then with 200 nM imatinib for additional 3 days (ACM-IM). Cell viability was analyzed by MTT assay. (D) The same experiments as described above were performed. Apoptotic cells were stained with annexin V-FITC and PI and analyzed by flow cytometry. Values are mean ± SEM from three experiments. ^*^, *p*<0.05 *versus* untreated control. ^#^, *p*<0.05 *versus* ctrl-shRNA/ACM-IM (C and D).

The p38MAPK inhibitor SB202190 significantly reduced ACM/imatinib sequential treatment-mediated growth inhibition (Fig. 6A) and apoptosis induction (Fig. 6B, C). Growth inhibition (Fig. 6D) and apoptosis induction (Fig. 6E, F) in ACM/imatinib sequential treatment were also reduced in K562/p38α(AF)1 cells compared to K562/mock cells. Moreover, p38MAPK knockdown was performed in K562 cells using shRNA plasmids. After transfection with shRNA plasmids for 3 days, the shRNAs successfully decreased p38MAPK level by approximately 90% (Fig. 7A). The p38MAPK knockdown reduced ACM-induced erythroid differentiation compared to control shRNA cells (Fig. 7B). The shRNA-mediated knockdown of p38MAPK significantly reduced ACM/imatinib sequential treatment-induced growth inhibition (Fig. 7C) and apoptosis (Fig. 7D). Those results suggest that erythroid differentiation appears sufficient to sensitize K562 cells in response to imatinib.

## Discussion

CML stem/progenitor cells are drug-insensitive, and imatinib fails to eliminate these cells [8], [9], [27]. The inability to treat successfully the disease is probably due to the survival of quiescent CML stem cells. Differentiation therapy provides an alternative treatment approach for cancer stem/progenitor cells. Here, the CML cell line K562 was used as a model to evaluate the differentiation induction treatment scheme. Our results showed that ACM differentiated cells were sensitized to imatinib and resulted in growth inhibition and apoptosis induction by modulating the down-regulation of Bcr-Abl, Mcl-1, and Bcl-xL, as well as the activation of the caspase-3.

ACM is an antitumor drug and is used to treat solid tumors, lymphomas and leukemias [10], [11], [12]. However, its high toxicity makes its clinical application still limited. Previous studies showed that a subtoxic concentration (20 nM) of ACM induced the erythroid differentiation of K562 cells [28]. It is worth noting that this subtoxic concentration of ACM used in differentiation studies was 50–200 times lower than the plasmatic concentration found in patients [29]. Sundman-Engberg et al. [30] reported that there are up to 85% of normal bone marrow cells can be grown in 5 nM ACM-containing medium. In the present study, we used a subtoxic concentration (5 or 10 nM) of ACM which mediated cell differentiation but not growth inhibition and apoptosis. Sequential treatment with imatinib in K562 cells with this low dose of ACM may have the potential to reduce toxicity and side effects, as part of differentiation therapy of CML.

Several antitumor compounds can be used as differentiation inducers for erythroid differentiation of CML cell lines. These compounds, such as MEK1/2 inhibitor PD184352 [31], histone deacetylase inhibitor SAHA [32] and morpholine derivative of doxorubicin [33], in combination with a subtoxic concentration of imatinib had synergistic effects. As single treatments, these differentiation agents like ACM alone treatment had no effect on cell death. However, our own work indicates that sequential treatment with ACM followed by a subtoxic concentration of imatinib strongly induced growth inhibition and apoptosis. This ACM/imatinib sequential treatment was more cytotoxic than simultaneous co-treatment with ACM and imatinib. Thus, regardless of the different treatment approaches, co-treatment or sequential treatment, induction of erythroid differentiation can markedly increase CML cells sensitivity to imatinib. In addition, a rational drug combination or sequential treatment can cause synergistic cytotoxicity effects and reduce individual drug-related side effects due to lowered doses of drugs.

Several studies reported that patients treated with imatinib still expressed Bcr-Abl despite inhibition of this kinase activity in CML stem/progenitor cells [34], [35] and in vitro study [36]. An effective strategy to reduce Bcr-Abl activity may be through the inhibition of Bcr-Abl expression level [37], [38], [39]. Our studies showed that sequential ACM/imatinib treatment down-regulated the expression level of the Bcr-Abl protein along with apoptosis. These findings suggest that the down-regulation of Bcr-Abl expression may be participate in the inhibitory activity of differentiation induction treatment in CML cells. The mechanism for the down-regulation of Bcr-Abl by ACM/imatinib sequential treatment needs further exploration. In addition to decreasing Bcr-Abl expression, the ACM/imatinib sequential treatment effectively decreased Mcl-1 and Bcl-xL expressions in K562 cells. Mcl-1 and Bcl-xL are anti-apoptotic members of the Bcl-2 family. Mcl-1 has been identified as a Bcr-Abl-dependent target and survival factor in CML cells [40], [41], and its up-regulation has been shown to play an important role in resistance to apoptosis [42]. So far, we do not know whether Bcl-xL is a target of Bcr-Abl signaling. However, other studies reported that the down-regulation of Bcl-xL expression is involved in apoptosis of K562 cells [43]. Thus, ACM/imatinib sequential treatment-induced cytotoxicity of K562 cells is associated with the down-regulation of Bcr-Abl, Mcl-1, and Bcl-xL.

Two pathways of caspase activation during apoptosis have been identified. The first (extrinsic) pathway starts at the death receptors on cell membrane such as Fas. Caspase-8 is the key initiator caspase in the extrinsic apoptotic pathway [25], [44], [45]. In the second (intrinsic) pathway, various pro-apoptotic signals converge at the mitochondria level, inducing the translocation of cytochrome c into the cytosol [46]. Cytochrome c-mediated caspase-9 activation triggers the activation of the executioner caspase-3 that leads to cell death [44], [45]. In this study, neither the expression levels of Fas ligand/Fas system nor the expression level of procaspase-8 were affected by ACM/imatinib sequential treatment. However, the cytosolic accumulation of cytochrome c, the down-regulation of procaspase-9, and the processing of caspase-3, suggested that the caspase-9/-3 pathway may be contribute to the ACM/imatinib sequential treatment-induced apoptosis. This viewpoint was further verified by the addition of caspase-3 inhibitor or caspase-9 inhibitor.

Previous studies demonstrated that the p38MAPK signal pathway is important for promoting erythroid differentiation. For example, the p38α isoform was involved in developmental and stress-induced erythroid differentiation [20]. Butyrate [47], GTP [22] and activin A [23] induce erythroid differentiation of K562 cells through the p38MAPK pathway. In agreement with the previous study, ACM induction of erythroid differentiation by p38MAPK pathway was observed in the present work, and then the loss of this differentiation reduced to sensitize K562 cells to imatinib effects. Therefore, differentiation of K562 CML cells is a key event that leads cells to be strongly sensitive to the chemotherapeutic drug, imatinib, in which p38MAPK plays an important differentiation role.

In conclusion, these results indicate that differentiated K562 cells induced by ACM became more sensitive to imatinib. Further studies would investigate whether our findings can be extended to CML primary cells, especially CML stem/progenitor cells.
