# Correction: Aclacinomycin A Sensitizes K562 Chronic Myeloid Leukemia Cells to Imatinib through p38MAPK-Mediated Erythroid Differentiation

**DOI:** 10.1371/journal.pone.0186528

**Published:** 2017-10-11

**Authors:** Yueh-Lun Lee, Chih-Wei Chen, Fu-Hwa Liu, Yu-Wen Huang, Huei-Mei Huang

The affiliation for the first author is incorrect. Yueh-Lun Lee is affiliated with: Department of Microbiology and Immunology, School of Medicine, College of Medicine, Taipei Medical University, Taipei, Taiwan.
